# The Evaluation of a Rapid Syndromic Multiplex Meningitis/Encephalitis RT-qPCR MX-17 Panel

**DOI:** 10.3390/diagnostics15202629

**Published:** 2025-10-17

**Authors:** Naim Mahroum, Meltem Yashar, Feyza Nihal Ugur, Nefise Zulal Oz, Gozde Ulfer, Ayse Istanbullu Tosun, Mesut Yilmaz

**Affiliations:** 1 Department of Infectious Diseases and Clinical Microbiology, International School of Medicine, Istanbul Medipol University, Kavacık, Göztepe Mah, Atatürk Cd. No: 40, Istanbul 34810, Turkey; atosun@medipol.edu.tr (A.I.T.); myilmaz@medipol.edu.tr (M.Y.); 2 School of Medicine, Istanbul Medipol University, Istanbul 34810, Turkey; meltem.yasar@std.medipol.edu.tr (M.Y.); feyzanihalugurr@gmail.com (F.N.U.); oznefisezulaloz@gmail.com (N.Z.O.); 3 Department of Biochemistry, Istanbul Medipol Mega Hospital, Istanbul 34393, Turkey; gozde.ulfer@medipol.com.tr

**Keywords:** syndromic testing, multiplex PCR panel, meningitis, encephalitis, CNS infection

## Abstract

**Background/Objectives:** Meningoencephalitis (ME) is a life-threatening infectious disease; therefore, prompt and accurate diagnosis is lifesaving. Traditional diagnostic methods, such as culture, have several limitations related to sensitivity and specificity. Emerging multiplex ME-PCR panels are a comprehensive and rapid tool in a single test. The Bio-Speedy Meningitis/Encephalitis RT-qPCR MX-17 panel (Bioeksen R&D Technologies Inc., Turkey) enables testing for 17 targets. To evaluate the performance of the panel compared to clinical and CSF parameters. **Methods:** A total of 403 patients with a preliminary diagnosis of ME were reviewed between January 2019 and September 2023. Following revision, 72 patients with clinical, CSF, and laboratory findings were included. The tested panel was used to detect targeted pathogens in CSF samples. The 30-day survival rate and prolonged stay were analyzed. **Results:** The median CSF protein value was 59.5 mg/dL (14.2–1471 mg/dL) and glucose was 61.95 mg/dL (0.083–165 mg/dL). Forty-one (56.9%) ME panel results were positive, among which 38.9% (28) were viral and 19.4% (14) were bacterial. HHV-6 ranked first with a rate of 15.3%. The Bio-Speedy panel test results outperformed the CSF culture (*p* < 0.001). The correlation of the Bio-Speedy panel with impaired consciousness was statistically significant (*p* = 0.004). Six (8.3%) patients from the study group died within 30 days. **Conclusions:** Compared to traditional methods, Bio-Speedy panel was effective in identifying the causative agents of ME. The Bio-Speedy ME RT-qPCR MX-17 panel offers accurate detection of ME-causing pathogens. The implementation of the panel in clinical practice can impact patient management and improve outcomes.

## 1. Introduction

Meningoencephalitis (ME), inflammation of the brain parenchyma and meninges, is a life-threatening neurological disease caused by various infectious agents, including viruses, bacteria, or fungi. The diagnosis of this central nervous system (CNS) infection is often made at emergency departments (EDs) or primary care settings [1]. The clinical presentation of ME is highly variable, depending on patient age and medical background, rendering it challenging to differentiate between viral and bacterial etiologies or other diagnoses [2]. Notably, patients with bacterial meningitis may present without the classic signs of the disease, increasing the risk of misdiagnosis [2,3].

The severity of ME ranges from self-limited viral meningitis to bacterial meningitis and viral encephalitis, which are associated with substantial morbidity and mortality [4,5]. Even after the acute disease phase, patients may experience long-term sequelae that significantly affect their quality of life, such as loss of vision, hearing problems, seizures, and cognitive impairment. These complications impose a considerable burden on the healthcare system, encompassing both acute care and long-term follow-up and rehabilitation [6]. Therefore, early laboratory diagnosis and prompt treatment with appropriate antimicrobial agents are crucial to prevent neurological sequelae and reduce mortality [7,8].

Traditionally, ME is diagnosed through microbiological analysis of cerebrospinal fluid (CSF) samples by microscopy, bacterial culture, and antigen assay, alongside the identification of viral pathogens using single-plex real-time polymerase chain reaction (PCR) techniques [9]. Except for the PCR technique, traditional methods often have limitations in terms of sensitivity, specificity, and turnaround time [10]. To overcome these limitations, multiplex molecular technique targeting multiple viruses, bacteria, and fungi simultaneously via utilizing a single PCR panel has been developed as an alternative to existing methods. The concerned panels, well-known as syndromic testing, have gained popularity for diagnosing infectious diseases across numerous sample types, including CSF, blood, respiratory, and gastrointestinal specimens [11,12]. The Bio-Speedy Meningitis/Encephalitis RT-qPCR MX-17 Panel (Bioeksen R&D Technologies Inc., Sarıyer, Turkey) is a one-time reverse transcriptase and real-time PCR test used for the qualitative detection of viral, bacterial, and fungal agents in CSF. This panel is increasingly integrated into routine hospital diagnostics alongside conventional methods. In our current study, we aimed to evaluate the diagnostic performance of this panel.

## 2. Materials and Methods

Our study is a retrospective cohort review; therefore, informed consent was not deemed necessary by the institutional review board.

### 2.1. Selection of Participants

**Figure 1:**
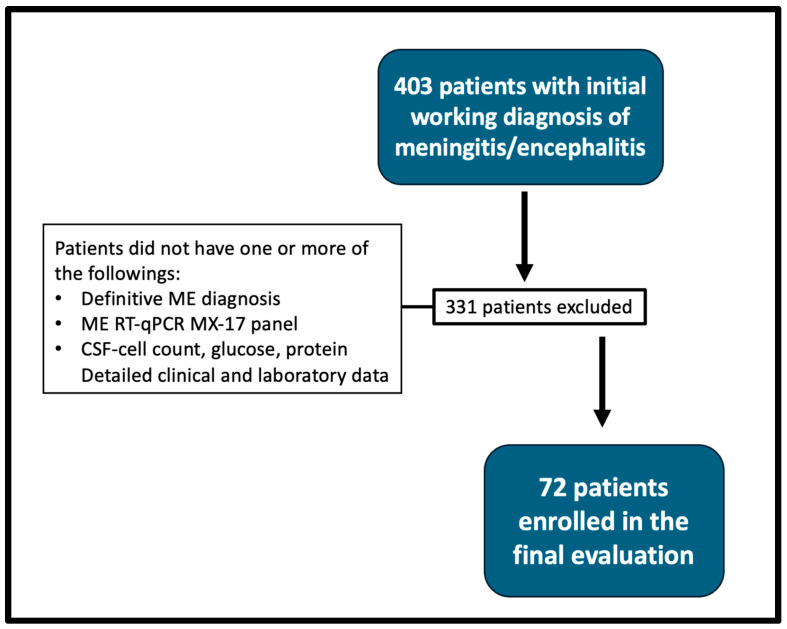
Flowchart of patient selection. ME—meningoencephalitis; CSF—cerebrospinal fluid; RT—reverse transcriptase; qPCR—quantitative polymerase chain reaction.

During the period from January 2019 to September 2023, 403 patients with ME findings who were admitted to Medipol Mega University Hospital were reviewed. Any participants who did not have one or more of the following: a definitive ME diagnosis, Meningitis/Encephalitis RT-qPCR MX-17 panel, CSF cell count, CSF glucose, CSF protein, and/or detailed clinical and laboratory data were excluded from our evaluation. Based on these criteria, 72 patients were eligible for the study (Figure 1). The patients selected were from the following departments of the hospital: emergency medicine, internal medicine, neurology (outpatient and inpatient sections), infectious diseases and clinical microbiology, hematology, neonatology, pediatrics and pediatric infectious diseases, and anesthesia-reanimation. The Bio-Speedy panel was utilized to detect targeted pathogens in the CSF samples, which were used as part of the diagnostic process by the department of infectious diseases and clinical microbiology in our hospital. The 30-day survival rate and prolonged stay were also calculated for all participants.

### 2.2. Cerebrospinal Fluid Culture

Lumbar puncture was conducted to obtain a minimum of 0.2 mL CSF, but larger volumes (typically 1–2 mL) were obtained for the full panel of tests, including culture, PCR, and biochemical analysis. Within two hours after collection, the sample was transferred to the microbiology laboratory and stored in a sterile, labeled vial. Culture and multiplex PCR were then performed. Each sample was taken separately into a sterile container for microbiological analysis. Conventional methods were used for culturing. A single culture media preparation was prepared from each sample using a 10 μL standard loop to inoculate on 5% sheep blood agar (Becton Dickinson, Oxford, UK) and ChromAgar Orientation Agar (Becton Dickinson UK). Culture media preparations were incubated at 37 °C for 24 h. The diagnostic process was performed according to microbiological instructions and guidelines [13].

### 2.3. Biochemical Analysis

A white blood count (WBC) from the CSF, alongside blood WBCs were analyzed by the flow cytometry method on the SYSMEX XN-1000 hemogram device (Osaka, Japan). CSF protein was analyzed by the turbidimetric method on a Roche Cobas c 503 autoanalyzer (Tokyo, Japan). CSF glucose was analyzed by the enzymatic method using a Roche Cobas c 503 autoanalyzer (Tokyo, Japan).

### 2.4. Bio-Speedy Meningitis/Encephalitis RT-qPCR MX-17 Panel

As mentioned earlier, the CSF samples were collected in a sterile manner. Subsequently, the nucleic acid extraction process was carried out using a magnetic bead-based automated Zybio Nucleic Acid Extraction System (Zybio, Chongqing, China). For nucleic acid extraction, 200 μL CSF sample and 15 μL Proteinase K were loaded into the cartridge, and the real-time PCR process was conducted by the Bio-Speedy Meningitis/Encephalitis RT-qPCR MX-17 Panel (Bioeksen, Istanbul, Turkey) using the nucleic acid obtained after the 9 min protocol. Result interpretation and automatic reporting process were completed in less than 1 min using the Sigmoida software v8.6REV.144 (Bioksen AR GE Teknolojileri A.Ş., Istanbul, Türkiye). The panel used for assessment is available in strip and tube formats. To perform the test, 0.1 mL of CSF was required. The test combines first-strand cDNA synthesis and subsequent PCR in a single reaction tube. The panel identifies 17 target pathogens (9 viruses, 6 bacteria, and 2 fungi): Herpes simplex virus 1 and 2 (HSV-1, HSV-2), Human Herpesvirus 6 (HSV-6), Human Herpesvirus 7 (HSV-7), Human Herpesvirus 8 (HSV-8), Varicella Zoster Virus (VZV), Enterovirus, Human parechovirus (HPeV), Cytomegalovirus (CMV), *Cryptococcus neoformans/gattii*, *Listeria monocytogenes*, *Streptococcus pneumoniae*, *Neisseria meningitidis*, *Streptococcus agalactiae*, *Haemophiles influenzae*, and *Escherichia coli* K1. The panel allows the achievement of RT-qPCR results in 60 min with a sensitivity of 100.00% and specificity of 98.04% [14]. Therefore, in terms of timing, the nucleic acid extraction was performed using a 9 min protocol, and then amplification, results were automatically interpreted by the Sigmoida software in less than 1 min. Finally, the overall turnaround time of the assay, from sample to final result, was approximately 60 min.

### 2.5. Analysis

The following data were obtained retrospectively from the patients’ files: demographics; clinical symptoms and signs such as fever, consciousness status, headache, and neck stiffness; as well as the 30-day discharge status of patients. CSF culture and Bio-Speedy panel results, CSF cell count, CSF glucose and protein values, blood profile, serum glucose, and CRP levels were all collected and arranged in Excel files. Following data collection, all the parameters were analyzed using Pearson’s chi-square test on SPSS v29. *p*-values of ≤0.05 were deemed statistically significant.

## 3. Results

A total of 72 patients met the inclusion criteria (Figure 1). The median age was 25 years (range 0–81), and 48 (66.7%) were males. Nineteen (26.4%) of the patients were immunocompromised. The most common clinical findings were altered consciousness in 41 (56.2%), fever of ≥38 °C in 30 (41.1%), headache in 16 (21.9%), and neck stiffness in 14 (19.2%).

In terms of laboratory findings, the median CSF protein value was 59.5 mg/dL (16.8–887.3 mg/dL, IQR), and the median CSF glucose value was 61.7 mg/dL (0–123 mg/dL, IQR). Glucose values between 50 and 75 mg/dL were considered normal.

Of the 66 available CSF cultures, 5 (7.5%) were positive. In contrast, the Bio-Speedy Meningitis/Encephalitis RT-qPCR MX-17 panel detected pathogens in 41/72 cases (56.9%) (Table 1). Among these, 28 (38.9% of total; 68.3% of positives) were viral, and 14 (19.4% of total; 34.1% of positives) were bacterial (Table 2), reflecting one additional bacterial detection due to coinfection. Blood cultures were positive in four patients (5.5%). A direct comparison of the diagnostic methods is shown in Table 3.

**Table 1:** Pathogens detected by Bio-Speedy^®^ Meningitis/Encephalitis RT-qPCR MX-17 Panel.

	Bio-Speedy^®^ ME RT-qPCR	CSF Culture	Blood Culture	Notes
**Viral pathogens**	Total: 28	-	-	Coinfected patients
Herpes simplex virus 1	5	N/A	N/A	
Human Herpesvirus 6	11	N/A	N/A	1 patient coinfected with Enterovirus
Human Herpesvirus 8	1	N/A	N/A	
Varicella Zoster virus	3	N/A	N/A	
Enterovirus	7	N/A	N/A	1 patient coinfected with HHV-6
Cytomegalovirus	1	N/A	N/A	
**Bacterial pathogens**	Total: 14	Total: 5	Total: 4	Coinfected patients
*Listeria monocytogenes*	3	1	2	
*Streptococcus pneumoniae*	5	3	1	1 patient coinfected with H. influenzae
*Neisseria meningitidis*	1	0	0	
*Streptococcus agalactiae*	2	0	0	
*Hemophilus influenzae*	1	0	0	Only detected in 1 coinfection with S. pneumoniae
*Escherichia coli* K1	2	1	1	

**Table 2:** Patient characteristics and survival for each subgroup were assessed. IQR* interquartile range, ME** meningoencephalitis.

	Viral	Bacterial	Aseptic Meningitis	*p* Value
Multiplex PCR analysis of 72 patients				
Patients (*n*)	28	14	30	
Age, median (IQR*)	19 (0–81)	4 (0–74)	10 (0–72)	
Gender (%)				0.79
● Female	41.4	6 (46.2)	6 (20)	
● Male	58.6	7 (53.8)	24 (80)	0.200
Laboratory Findings:				
CSF WBC				
● ≤5*n*	11	1	0	
● >5*n*	17	12	30	
Mean	424.8 ± 889.4	1903.6 ± 3960	1259.6 ± 4618.2	0.101
Median (IQR*)	66 (1–4221)	315 (4–14,963)	30.5 (9–25,085)	
CSF glucose level				0.189
● <50 mg/dL	9	8	8	
● 50–75 mg/dL	13	2	13	
● >75 mg/dL	6	3	9	
Mean	66.8 ± 29.3	42.8 ± 43.7	61.7 ± 30.1	
Median (IQR*)	56.8 (33.9–165)	29.9 (0.083–157)	62.8 (0.123–140)	
CSF protein level	69 ± 50	228.3 ± 224.6	162.6 ± 300	0.282
Median (IQR*)	55.5 (14.2–200)	143.9 (35.2–531)	59.5 (16.8–887.3)	
CRP level	46.2 ± 77.5	137.2 ± 136	35.8 ± 70.9	0.391
Median (IQR*)	15.2 (0.142–329.5)	85 (6.18–41.3)	12.45 (0.20–83.8)	
Clinical Features:				
Fever (Mean ± SD (°C))	37.22 ± 0.9	37.6 ± 1.3	37.3 ± 0.8	0.95
Impaired consciousness *n* (%)	9 (32.14)	10 (76.9)	21 (70)	0.004
Neck stiffness *n* (%)	6 (21.43)	4 (30.8)	2 (6.6)	0.113
Headache *n* (%)	11 (39.3)	1 (7.1)	4 (13.3)	0.017
Outcomes:				
Prolonged stay in the hospital (>7 days) *n* (%)	13 (46.4)	6 (46)	6(20)	0.067
**Among prolonged stays:**				
Patients age *n* (%)				
● <1 year	4 (30.8)	2 (33.3)	-	
● 1–17 year	4 (30.8)	2 (33.3)	4 (66.6)	
● 18–64 year	3 (23)	-	2 (33.3)	
● 65 or older	2 (15.4)	2 (33.3)	-	
Underlying condition other than ME** *n* (%)	8 (28.5)	2 (14.2)	1 (3.3)	
30-day survival *n* (%)	26 (92.8)	12 (85.7)	28 (93.3)	0.594

**Table 3:** Comparison of the positive results according to various diagnostic methods used.

Method	Positive Cases, *n* (%)	Viral, *n* (%)	Bacterial, *n* (%)
Bio-Speedy PCR (*n* = 72)	41 (56.9)	28 (38.9)	14 (19.4)
CSF culture (*n* = 66)	5 (7.5)	-	6 (9.1)
Blood culture (*n* = 72)	4 (5.5)	-	4 (5.5)

According to the ME panel results, HHV-6 was the most frequently detected pathogen, identified in 11/72 patients (15.3%). Among PCR-positive cases, HHV-6 accounted for 11/41 (26.8%), and among viral causes specifically, 11/28 (39.3%). Enterovirus was detected in 7/72 patients (9.7%), and Streptococcus pneumoniae in 5/72 (6.9%). Two patients had coinfections: one with HHV-6 + Enterovirus, and another with *S. pneumoniae* + *H. influenzae*. Pathogen counts include all detected organisms.

**Figure 2:**
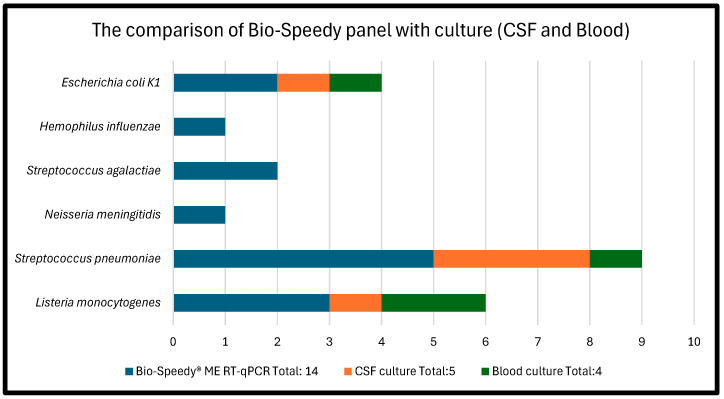
Comparison of the performance of Bio-Speedy panel with CSF and blood cultures.

Thirty patients (41.6%) remained without an identified etiology. One patient who initially had negative PCR, had later developed positive CSF culture (*S. pneumoniae*), so he was removed from the aseptic group; two had no culture available. When compared to culture methods (blood and CSF), the Bio-Speedy ME panel test results outperformed CSF culture (*p* < 0.001) (Figure 2).

Thirteen patients (18.1%) had a prolonged hospital stay (>7 days). Six patients (8.3%) died within 30 days: two with bacterial meningitis, two with viral encephalitis, and two with aseptic meningitis. There was one patient with underlying leukemia in each of the aseptic, bacterial, and viral groups (three patients total); all three of these patients died. The Bio-Speedy PCR positivity did not correlate with higher mortality, but PCR positivity was significantly associated with impaired consciousness (*p* = 0.004) (Table 2).

## 4. Discussion

ME is a serious infectious disease affecting the CNS. The prognosis is highly dependent on the time required for diagnosis and initiation of treatment. The quicker the approach, the better the outcomes [15]. Thus, diagnostic methods that shorten the time to diagnosis using a single CSF sample from patients suspected of ME are extremely valuable and potentially lifesaving. In this regard, the Bio-Speedy Meningitis/Encephalitis RT-qPCR MX-17 panel, evaluated in our study, demonstrated both reliable and accurate results.

The standard of care for patients with suspected ME includes CSF analysis, Gram staining, bacterial culture, and viral PCRs (mainly for EV and HSV-1/2) [16]. CSF culture is the gold standard diagnostic method for bacterial meningitis; however, final results are typically available only after 48 h [17]. The most common form of ME (one- to two-thirds of cases) is aseptic, defined as the absence of bacterial growth in cultures, and is frequently caused by viruses [18,19]. Hence, ME can remain undiagnosed, especially in viral cases [20]. Enteroviruses (Coxsackie or Echovirus) are the most common cause of viral meningitis in all age groups [21]. Additionally, human herpes viruses (HHVs), adenovirus, influenza, parainfluenza, and mumps can also cause ME [22,23]. The Bio-Speedy Meningitis/Encephalitis RT-qPCR MX-17 panel not only enables a rapid assessment within 60 min but also allows a comprehensive and simultaneous evaluation of multiple pathogens, including six viruses, in a single test. In our cohort, 38.9% of patients had a viral etiology of ME, 19.4% had bacterial ME, while 41.6% remained undetermined. The panel was also able to identify coinfections, which, although rare, can be encountered in clinical practice and are often missed by conventional methods. In our study, two patients were infected with more than one pathogen (one with two viruses, the other with two bacteria). The ability of the ME panel to diagnose such infections further supports its role in identifying pathogens that might be overlooked due to overlapping clinical features [24].

The performance of ME panels has been evaluated in several studies. For instance, a panel capable of identifying 11 pathogens with 100% sensitivity and 98.5% specificity was previously reported by Sapra and colleagues [25]. Another paper assessing a multiplex PCR panel (FilmArray) for CNS infections, capable of detecting 16 bacterial, viral, and fungal agents, showed comparable results to standard diagnostic methods (culture and viral PCR) [26]. Notably, the FilmArray panel was able to detect pathogens in culture-negative samples. The Bio-Speedy panel evaluated in our study detects more pathogens, with the exception of *Mycobacterium tuberculosis*. In our cohort, the most common viral agent detected was HHV-6, while the most common bacterial agent was *S. pneumoniae*. In fact, *S. pneumoniae* is the leading cause of bacterial meningitis in children older than one month and in all adult age groups, with high morbidity and mortality [27,28]. In turn, HHV-6, a causative agent of encephalitis, is prevalent during infancy and early childhood [29], while in adults it is primarily seen in immunocompromised patients [30,31]. In our study, HHV-6 was detected in eleven patients: seven children and four immunocompromised adult patients.

CSF parameters are also valuable in the preliminary diagnosis of ME, particularly in culture-negative cases where viruses, other bacteria, or *M. tuberculosis* can be the causative agents. Pleocytosis of CSF is a sensitive marker of inflammation and infection in both adults and infants [32,33,34], though it may also be caused by CNS tumors, non-infectious neurological diseases, or extra-CNS infections [35]. Leukocyte counts above 100/μL are mainly associated with CNS infection. Typically, bacterial meningitis is characterized by higher counts (1000 to 5000/μL) compared to viral etiologies (usually <500/L) [36]. In our cohort, CSF protein, glucose, and leukocyte patterns were generally consistent with these expected profiles, with bacterial meningitis cases showing higher protein and lower glucose values, while viral cases tended to present with milder abnormalities. A key consideration in this aspect is that the reference range of CSF parameters varies in infants. In a large cohort of preterm newborns with meningitis, WBC, protein, and glucose levels in the CSF showed poor predictive value [37]. Similarly, another study of more than 1000 CSF samples from 948 pediatric patients found that relying on pleocytosis or other abnormal CSF parameters to guide ME panel testing led to frequent misdiagnoses [38]. The findings underscore the importance of ME panels in this age group.

When compared to microbiological analysis of CSF, Gram staining has been reported to have a sensitivity ranging between 10 and 93% and nearly 100% specificity, depending on the microorganism and the population examined [32]. Culture sensitivities usually fall around 70–90%, with almost 100% specificity. However, antibiotic treatment can markedly reduce sensitivity. For example, prior antibiotic treatment reduced the sensitivity of CSF Gram stain and culture to 40–60% and to below 50%, respectively [39]. Reduced sensitivity prior to lumbar puncture and/or missing the 72 h testing window hinder timely diagnosis and treatment. In contrast, HSV PCR results remained positive for at least 6–7 days after disease onset, even with acyclovir therapy [9]. The Bio-Speedy panel in this case outperformed both Gram stain and culture, with a sensitivity of 100% and specificity of 98.04%. Moreover, due to its PCR nature, it is less affected by prior antibiotic/antiviral treatment.

Our findings highlight the potential value of using ME panels combined with CSF culture as complementary diagnostic tools. By detecting several pathogens, including those that are challenging to culture or require specialized techniques, the panel enhances diagnostic efficiency by enabling rapid, accurate, and simultaneous detection. The use of ME panel has already been well received, with expectations of reducing diagnostic time, CSF volume required, empirical antibiotic use, and overall expenses [40,41,42]. To sum up, key advantages of ME panel include detecting pathogens in less than one hour, the ability to detect pathogens without requiring viable organisms, and being less affected by antibiotic treatment than culture methods [42]. Despite the positive attributes of ME panels, antimicrobial susceptibility testing still requires culture. Moreover, ME panels do not detect nosocomial meningitis pathogens which can develop after trauma or surgery and can only be detected by culture (*Klebsiella pneumoniae, Acinetobacter, Pseudomonas, and methicillin-resistant Staphylococcus aureus*) [43].

Beyond diagnostic accuracy, rapid molecular testing has an important clinical implication in therapeutic decision-making. Early pathogen identification by the ME panel in our cohort facilitated adjustment of treatment in several cases. For instance, the detection of viral etiologies (38.9% of patients) supported stepping down from empirical broad-spectrum antibiotics, thereby reducing unnecessary exposure. Similarly, rapid confirmation of bacterial pathogens such as *S. pneumoniae* allowed therapy to be tailored appropriately. Moreover, the identification of HHV-6 in immunocompromised patients contributed to the early initiation of antiviral therapy. These observations support the fact that the application of ME panels does not only expedite diagnosis but also directly informs therapeutic measures, promoting both antimicrobial stewardship and patient safety.

One final note before addressing the study limitations concerns the number of aseptic meningitis cases in which no etiological agent was identified despite being clinically diagnosed. In our cohort, this proportion reached 41.6%, which may appear high; however, similar or even higher rates have been reported in the literature [44,45]. We fully agree that this remains a significant challenge, as the undetected pathogen may be one not included in the ME panel. This underscores both the current gaps in available diagnostic tools and the clear need for future refinement of diagnostic panels for meningitis and encephalitis.

Our study has several limitations which can be summarized in the following points. First, this was a single-center study, and the small sample size limits the generalizability of the results. Conducting multicenter and prospective studies on the benefits of incorporating molecular methods in routine practice may provide more robust data for patient management. Second, the retrospective design of the study made it challenging to collect uniform data across participants. Nevertheless, as molecular testing was our main focus, all participants underwent these tests, minimizing issues related to missing data. Moreover, due to the fact that viral ME, such as Enterovirus, are mainly asymptomatic and we selected only symptomatic patients, our study may pose a possible selection bias stemming from the inclusion criteria. Third, not all the targeted microorganisms of our ME panel were encountered during the study period, preventing full evaluation of its performance. However, given the panel’s reliable performance for the microorganisms detected, we expect similar diagnostic accuracy for other pathogens included.

## 5. Conclusions

Prompt diagnosis and treatment of ME are crucial, as time plays a critical role. The Bio-Speedy Meningitis/Encephalitis RT-qPCR MX-17 panel enables single-run, simultaneous detection of numerous relevant and commonly encountered ME-causing pathogens, with results available within 60 min. Its performance is comparable to, and in many aspects superior to, standard diagnostic methods across bacterial, viral, and fungal pathogens in all age groups. The implementation of this panel in clinical practice contributes to faster diagnosis, earlier initiation of appropriate treatment, and in many cases, timely de-escalation or adjustment of empirical therapies.

## Data Availability

The original contributions presented in this study are included in the article. Further inquiries can be directed to the corresponding author.
